# A novel guidewire technique for endoscopic mucosal resection of ileocecal valve lesions

**DOI:** 10.1055/a-2697-2266

**Published:** 2025-09-22

**Authors:** Jun Young Kim, Sunil Gupta, Clarence Kerrison, Brian Lam, Hasib Ahmadzai, Nicholas G. Burgess, Michael J. Bourke

**Affiliations:** 1 8539 Department of Gastroenterology and Hepatology, Westmead Hospital, Sydney, Australia; 2 216997 The University of Sydney Westmead Clinical School, Sydney, Australia

Large nonpedunculated colorectal polyps (LNPCPs) of the ileocecal valve (ICV) are challenging to resect endoscopically due to their difficult location and limited access. However, endoscopic resection can be highly successful and surgeries avoided 1 . A retrospective cohort study of 118 ICV lesions followed over 18 years demonstrated clinically successful endoscopic mucosal resection (EMR) in only 76.3%, with recurrence observed in 16.5% at first follow-up 2 . Technical advances such as margin thermal ablation with soft-tip snare coagulation (STSC), cold avulsion with adjuvant STSC of nonlifting areas, and assessment of deep mural injury have significantly improved outcomes such that Vosko et al. reported clinically successful EMR in 93.9% with a recurrence rate of 4.6% 3 . Notably, clinical success declines with increasing terminal ileal extension and greater circumferential ICV involvement. Deep terminal ileal involvement (≥10 mm) was associated with EMR failure requiring surgery 3 . Therefore, here we report here a novel adjunct technique in which the use of an ultrastiff guidewire improves access to the terminal ileum, thereby facilitating safe and precise endoscopic resection, particularly in the terminal ileum ( Video 1 ).

Guidewire-assisted endoscopic mucosal resection of a fully circumferential large, nonpedunculated colorectal polyp of the ileocecal valve with terminal ileal extension.Video 1

A 73-year-old man was referred for resection of a 60-mm Paris 0–IIa, nongranular, fully circumferential ICV lesion with ileal extension. EMR was challenging due to the lesion location and difficult access ( Fig. 1 **a, b** ). Conventional maneuvers such as transabdominal pressure and position change did not improve visualization. The ileal extent of the lesion could not be accurately determined, nor could the ileum be sufficiently maneuvered. An ultrastiff guidewire was therefore passed through the scope into the ileum. This opened the terminal ileum significantly and allowed access to accurately visualize the extent of the lesion and provide a greater working field ( Fig. 1 **c** ). Safe and precise sequential EMR was made possible and furthermore facilitated effective margin thermal ablation, and defect assessment to rule out deep mural injury ( Fig. 1 **d** ). There were no complications from the procedure, and the histology returned a diagnosis of tubular adenoma with low-grade dysplasia ( Video 1 ).

**Fig. 1:**
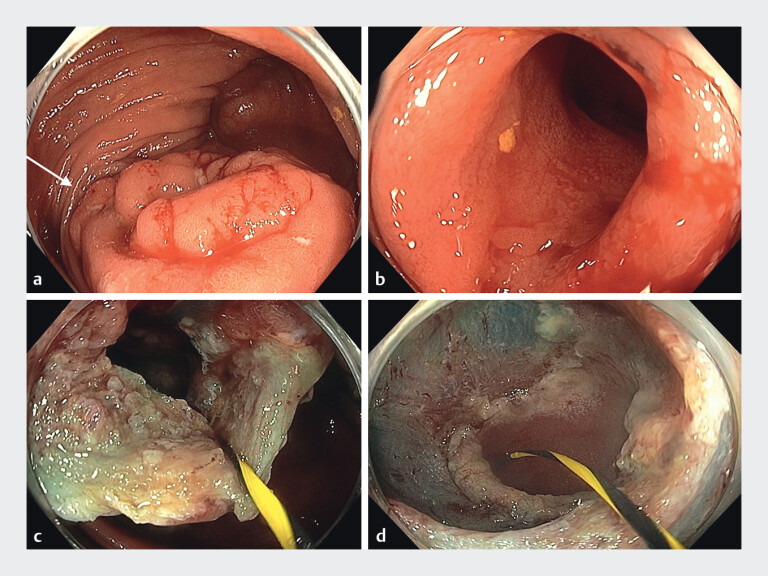
**a** Endoscopic view of the 60-mm, fully circumferential Paris 0–IIa, nongranular ileocecal valve lesion. **b** Access into the terminal ileum was difficult. **c** Access improved with the insertion of an ultrastiff guidewire. **d** Endoscopic appearance after resection and margin thermal ablation.

We demonstrate a novel technique of how a through-the-scope ultrastiff guidewire can effectively facilitate EMR of difficult ICV lesions, particularly in those extending into the terminal ileum.

Endoscopy_UCTN_Code_TTT_1AQ_2AD_3AD

**Correction**

**Correction: A novel guidewire technique for endoscopic mucosal resection of ileocecal valve lesions** Kim Jun Young, Gupta Sunil, Kerrison Clarence et al. A novel guidewire technique for endoscopic mucosal resection of ileocecal valve lesions. Endoscopy 2025; 57: E1094–E1095, doi:10.1055/a-2697-2266 In the above-mentioned article the author name of Hasib Ahmadzai has been corrected. This was corrected in the online version on September 24, 2025.

## Notes

Endoscopy E-Videos https://eref.thieme.de/e-videos
*E-Videos* is an open access online section of the journal *Endoscopy* , reporting on interesting cases and new techniques in gastroenterological endoscopy. All papers include a high-quality video and are published with a Creative Commons CC-BY license. Endoscopy E-Videos qualify for HINARI discounts and waivers and eligibility is automatically checked during the submission process. We grant 100% waivers to articles whose corresponding authors are based in Group A countries and 50% waivers to those who are based in Group B countries as classified by Research4Life (see: https://www.research4life.org/access/eligibility/ ). This section has its own submission website at https://mc.manuscriptcentral.com/e-videos .

## Notes

Correction **Correction: A novel guidewire technique for endoscopic mucosal resection of ileocecal valve lesions** Kim Jun Young, Gupta Sunil, Kerrison Clarence et al. A novel guidewire technique for endoscopic mucosal resection of ileocecal valve lesions. Endoscopy 2025; 57: E1094–E1095, doi:10.1055/a-2697-2266 In the above-mentioned article the author name of Hasib Ahmadzai has been corrected. This was corrected in the online version on September 24, 2025.
