# Correction: A novel guidewire technique for endoscopic mucosal resection of ileocecal valve lesions

**DOI:** 10.1055/a-2707-8117

**Published:** 2025-09-24

**Authors:** Jun Young Kim, Sunil Gupta, Clarence Kerrison, Brian Lam, Hasib Ahmadzai, Nicholas G. Burgess, Michael J. Bourke

**Affiliations:** 1 8539 Department of Gastroenterology and Hepatology, Westmead Hospital, Sydney, Australia; 2 216997 The University of Sydney Westmead Clinical School, Sydney, Australia



In the above-mentioned article the author name of Hasib Ahmadzai has been corrected. This was corrected in the online version on September 24, 2025.

